# Methods of Clinical Coprology as a General Indication for Diet

**Published:** 1913-10

**Authors:** Rene Gaultier

**Affiliations:** PARIS. Ex-Chief of the Medical Clinic of the Hotel Dieu and Assistant Physician to the Beaujon Hospital


### Methods of Clinical Coprology as a General Indication For Diet

DR RENE GAULTIER, PARIS. Ex-Chief of the Medical Clinic of the Hotel Dieu and Assistant Physician to the Beaujon Hospital.

CLINICAL coprology has become, in recent years, the basis of a new semeiology of the digestive tract which no physician should ignore if he wishes his patients to profit by a rational calculated diet. In this article only the general principles of these methods and their applications to diet will be considered, the reader being referred for details to the author's *Precis de Coprologie Clinique,* published by Bailiiere, Paris, 1907.

### I.

Foods have a definite nutritive value and a determined digestibility, while individuals have a definite nutritive power and state of nutrition subject to modification from time to time. Both of these factors must be considered in establishing a diet. It is necessary to know the composition of a food with reference to its absorbable materials. Its digestibility depends upon the form in which it is administered, as raw or cooked in different ways, so that it is equally necessary to understand the modifications to which different forms of culinary preparation lead. There is no necessary connection between the digestibility and the theoretic nutritive value of a food, the most easily digestible foods not being always the most nourishing. The state of nutrition varies according to the individual and in the individual at different periods. It is measured by the general nutritive balance. The digestive capacity varies similarly and is measured by what may be termed the coprologic nutritive balance. It may be added that there is no fixed relation between the digestive power and the state of nutrition, the former depending on an organ, the alimentary canal, the latter on the organism as a whole. Books on dietetics give ample instruction in the nutritive value, digestibility and methods of preparation of foods but are silent as to the coprologic balance, for the reason that the examination of fæces has only recently emerged from the domain of pure science.

### II.

What are the methods of clinical coprology? How do they assist, what are their applications, general and particular, in dietetics?

These methods consist in a functional exploration of the digestive organs, being analogous to those with which we have become accustomed in the elaboration of the urinary molecules by the kidney (cryoscopy), to those which inform us as to the effective work of the gastric mucosa (gastric chemistry), in short, throwing light on the ingesta through the study of alimentary residue so that we may estimate their utilization and thus adapt to the individual digestive capacity a regime whose proximate principles correspond to the caloric requirements of the particular nutritive condition. As Ricardo Lynch has justly said, "A normal digestion is that which not only causes no trouble, but that in which the utilization of aliments is adequate and in harmony with the individual digestive processes." It is because the systematic examination of stools enables us to recognize this utilization that it is to be recommended to the use of the general practitioner.

### III.

Of what do the methods of clinical coprology consist? This is not the place to pass in review the different methods proposed. It seems more natural to allude rather to the methods which I have recommended in France since 1904, and as to which I can speak from experience and experimental research. We start from a logical test meal, designed to put into action the different digestive glands and thus test their functional power. It consists of

| | |
|---|---|
| White bread | 100 grams |
| Beef or mutton | 60 grams |
| Butter | 10-30 grams |
| Milk | 300-500 c.c. |
| Potatoes | 100 grams |

The meat is broiled rare, served with bread and butter. It should be cut into small bits and well chewed. The potatoes are boiled, mashed and prepared with milk and a little butter. The rest of the butter is eaten on the bread. This meal should be taken after a considerable fast after the next previous meal, hence, most conveniently, for breakfast. The delimitation of the fæces corresponding to the test meals is secured by the administration of three cachets of carmine powder, 0.30, at the beginning, middle and end of the test meal. The entire stool thus reddened is collected. If there is no hurry, it is well to precede the test meal by a milk diet for two days and, after 6 or 8 hours, resume the milk diet for another day, so that the reddened fæces stand out clearly between the grey or colorless ones, and so as to avoid the stench due to an ordinary mixed diet.

The fæces thus collected are examined as to duration of alimentary transit, general physical characteristics, and also microscopically, macroscopically, chemically and, if necessary, bacteriologically. From the coprologic syndrome thus obtained, certain conclusions may be drawn, to be considered later.

The duration of the alimentary transit informs us as to intestinal motility. A diagnosis cannot be established on this sign alone, but, with other signs, it assumes a real diagnostic value. It is very simple, requiring merely the noting of the time of appearance of the first and the last red stool after the test meal. Its normal limits are 26 to 40 hours, eliminating sources of error due to the quantity and quality of food by depending only on the results of the test meal. With the determination of the ratio between the weights of the dry and the fresh fæces, the duration of alimentary transit is a precise indication of the existence of diarrhœa or constipation. Really, the common notion regarding these two terms is most inaccurate. The mere presence of liquid and frequent stools cannot accurately determine a diarrhœa nor can that of hard and infrequent stools determine a constipation, since neither condition can inform us as to motility, secretion and absorption. The ordinary evidence of constipation or diarrhœa, based on the observation of the patient alone and interpreted by the physician on the say-so of his client, is inadequate. Such testimony should serve merely as a guide to the more attentive study of the fæces.

It is difficult to establish an arbitrary definition of constipation. Some persons in good health have a single daily passage; others have passages only every two or three days and appear not to suffer at all on this account. It would be an error to term them constipated. Masked constipation is also frequent: that is, the patient has a stool every day, regularly, but still the examination of the fæces corresponding to a given test meal, shows delay in the intestinal motor function. However, true constipation may exist without obvious trouble and is often unknown and neglected. Or, there may be true constipation while the patient believes himself to have a diarrrhœa on account of the irritation of the intestinal mucosa by indurated scybala.

One should not use the term constipation until, after giving a test meal with carmine, (1) the red stools do not appear until after a longer time than indicated above (26-40 hours) ; (2) or that they contain a higher percentage of dry matter than normal.

Conversely, the diagnosis of diarrhœa requires (1) that the red stools appear in a shorter time than normal; (2) that they contain a less proportion of dry matter than normal.

Aside from direct disturbances of motility, a prolongation of the alimentary transit may be due to deficient biliary secretion while a shortening of the transit suggests a deficiency of the pancreatic juice, of intestinal absorption. However, the lengthening or shortening of the alimentary transit may be due merely to trouble in the nervous system, as may be deduced from the absense of assimilable waste in the stools—with special reference to fats.

To determine the proportions of water and dry matter in the fæces, a small quantity of the stool collected after the test meal (small to insure thorough dessication in a reasonable time) is weighed in a tared capsule, and dried in a water (or sand) bath at 100. C., stirring occasionally. When dessication seems to be complete, the capsule is again weighed, then replaced on the water bath for a time and again weighed, till two consecutive readings are the same. The amount of water lost is found by comparing the weights of the fresh and dessicated sample. (Note—Percentages should be calculated on the net weight in each case, subtracting the tare of the capsule). The normal ratio in man, with the test meal, is about 68 per cent. water and 32 per cent. dry matter.

The general physical characteristics of fæces also afford some information. The total weight, with the normal intestinal function, after the test meal, should be about 100 grams, undried. A greater weight, without being of great importance by itself, indicates one of two things; a pathologic augmentation of waste products of the alimentary canal or incomplete digestion or absorption of ingesta, which can, theoretically, be verified by the macroscopic and microscopic or chemic examination of the fæces.

The normal consistence of the fæces should be firm. If liquid, or not homogeneous, pasty like mastic or pommade, further examination is required to locate the abnormality. The form also has a clinical interest. Instead of the normal sausage like moulding, the fæces may be crinkled, hard and dry, scybalous, as in mucomembranous enteritis with intestinal spasm. On the other hand, they may be soft, pasty, diffluent, like cow droppings, in certain forms of colitis with muscular atony. They may be ribbon-like or wiredrawn, in contraction of the rectum.

The odor may also aid the diagnosis. Considering that the test meal contains milk and, especially if it is preceded by a milk diet, the stools should have an odor that is scarcely disagreeable. A putrid, nauseating odor indicates putrefaction which further analysis will determine.

The macroscopic examination, according to Schmidt, constitutes the essential part of the fæcal analysis, but it does not seem to me to furnish very useful information except in conspicuously pathologic cases. The normal stool, after the test meal designated, should contain neither alimentary debris visible to the naked eye nor pathologic products such as blood and pus. To make the detailed examination of the stool, a small quantity is thoroughly rubbed up in a mortar with distilled water to secure homogenity and a thin layer in a Petri dish is examined over a black or a white background. With the aid of Koenig's table, various debris can be identified. (Note—It should be borne in mind that this article deals with the stools collected after the test meal. Otherwise, without abnormality, the stool may be very heterogeneous and may show large masses of vegetable debris.) While the normal stool is homogeneous, the pathologic stool includes particles larger than normal, including:

1. Alimentary residue:

*a.* Remains of connective tissue, yellowish-blue corpuscles of firm consistence, infilaments, indicative of failure of gastric digestion.

*b.* Remains of muscular tissue, small brownish fragments, like chips of wood, indicative of pancreatic insufficience.

*c.* Residue of potato, appearing as opalescent granules, indicative of failure of intestinal digestion.

*d.* Fatty residue, having a soft, gravy-like appearance, of yellowish white color, indicative of failure of the biliary and pancreatic secretions.

2. Pathologic products from the intestine:

*a.* Mucus, sometimes as yellowish-grey slime covering the fæcal mass and mixed with it, as is apparent when it is stirred with water, letting the material drip from the stirring rod.

*b.* Membranes. Sometimes there are fibrinous flakes, tubes, ribbons, or shreds with irregular borders.

*c.* Mucus. Sometimes there are little yellowish globules of gelatinous consistence, intimately mixed with the fæces, difficult to determine macroscopically from potato debris. The distinction between these forms is of the utmost clinical importance, for upon it depends the localization of an inflammatory alteration of the intestine. According to Nothnagel, mucus from the lower part of the large intestine is unmixed, more or less thick and transparent, while that from the upper part of the large intestine is mixed with the non-homogeneous fæces and that from the small intestine is scarcely apparent macroscopically and is intimately mixed with homogeneous fæces.

*d.* The establishment of pus is important clinically. With it may be mentioned the finding of tumor debris, which is rarely possible.

*e.* Calculi and concrements. The finding of enteroliths, coproliths, appendicular or biliary calculi, with the presence of blood or parasites, completes the pathologic macroscopic examination.

The microscopic examination is, without doubt, superior to the preceding. Solid masses, suspicious particles detected in the Petri dish macroscopically, or some of the homogeneous mass of fæces, is subjected to examination between slide and cover. Without the addition of any reagent, one notes:

*a.* Muscular fibres, more or less altered, recognizable by their yellow color, swollen appearance and transverse striations.

*b.* Elastic fibres, recognizable by their feeble contour and tortuous shape.

*c.* Yellow bodies of Nothnagel, disposed in heaps, which are considered to be blocks of coagulated albumin.

*d.* Yellow calcium soaps, with straight outlines, many cornered; also colorless magnesium soaps, otherwise similar.

*e.* Some starch cells of potato and bread.

In the pathologic stool one notes in addition:

*a.* Muscular fibres in greater abundance, indicative of failure of pancreatic secretion.

*b.* Connective tissue fibres, colorless wavy filaments, measuring several millimeters, rarely bifurcated, indicative of failure of gastric secretions.

*c.* Elastic fibres, voluminous filaments, transparent, highly refractive, wavy, bifurcating and anastomosing in a net work.

*d.* Residue of carbohydrates, particularly of bread and potato, recognizable by their starch granules and stained blue by a solution of I plus KI, indicating a disturbance of the small intestine.

*e.* Fatty residue in the form of drops of neutral fat, soluble in ether or chloroform, some amorphous, others shining, more or less tinged with yellow, blackened by osmic acid or colored orange by Soudan III. Fatty residue may also take the form of acicular crystals of fatty acids, heaped together like a pile of needles, or it may take the form of yellow soaps of calcium of polygonic contour. The greater or less abundance of neutral fats, fatty acids or soaps, and their relative proportions, indicate, respectively, biliary or pancreatic troubles.

In pathologic stools, one may also occasionally encounter red blood cells, sometimes leucocytes, more or less degenerated, indicating an ulcerative process in the intestine, or desquamated epithelial cells indicative of a catarrh. There must also be mentioned crystals of ammonio-magnesium phosphate, indicative of intestinal putrefaction; masses of calcium salts without exact significance but perhaps indicative of decalcification; crystals of cholesterine, whose persistence indicates exaggerated peristalsis; crystals of hæmatoidin, characteristic of intestinal hæmorrhage.

The Chemic examination should determine the following:

1. Reaction, determined with litmus paper is normally, after the test meal, neutral but is affected by many factors. A strict meat diet gives rise to an alkaline reaction, an exclusive carbohydrate diet to an acid reaction, as does a diet rich in fats. I have been able to establish the following two facts: (*a*) that prolonged alimentary transit plus acid reaction indicates motor deficiency of the small intestine; (*b*) that prolonged alimentary transit plus alkaline reaction indicates motor deficiency of the large intestine.

2. Biliary pigments, determined by adding to the stool mercuric chlorid in concentrated solution, are normally colored red, indicating that they are in the form of hydrobilirubin. Pathologically a green color occurs, indicative of the unreduced form, bilirubin, changed in biliverdin by oxidation.

3. The relation of the weight of dry matter to the fresh stool has already been considered.

4. The investigation of the utilization of fats, qualitative and quantitative, remains to be considered. This is, in our opinion, of the highest importance as, alone, it permits an exploration of the function of the digestive canal and a recognition of the functional activity of its various parts and tributaries. This subject was treated in the author's inaugural dissertation in 1905. It is too lengthy to be considered here in detail.

5. Finally, the investigation of the pigments of the blood by the classic reaction of Weber, guaiac and hydrogen peroxid, or one of its numerous modifications.

The bacteriologic examination completes the coprologic analysis. We know that the intestinal flora is in relation with the culture medium. The colon bacillus is always present. In pathologic stools, aside from specific microbes such as the tubercle or diphtheria bacillus, etc., there is an exaggerated growth of many types indicative of intestinal putrefaction, or fermentation more or less limited or combined. The bacteriologic examination is made quite simply by the ordinary staining methods, after a double centrifugalization of a small mass of fæces, first with pure water, then with 90 per cent. alcohol.

Now, what are the practical applications of these coprologic methods in the art of dietetics? I shall not enter into the diagnostic information which they may furnish, as pointing toward insufficient mastication, bad gastric digestion, abnormal pancreatic or biliary secretion, or trouble with the intrinsic intestinal glands, either in the academic sense of establishing a diagnosis or in their numerous practical indications, but merely point out the general utility of these methods in establishing a rational dietary. The systematic examination of the fæces, particularly the study of the utilization of the fats enables us to judge the degree of utilization of foods and to calculate the ration sufficient for the individual. If we consider only the general nutritive balance, in tuberculosis, for example, nutrition may be deficient because the individual capacity is not determined. When fats are administered in increasing quantity to a tuberculous patient, one may notice at first that he excretes less nitrogen and takes on weight. Hence, it might be concluded that fatty nourishment is the one of choice for tuberculosis. But, if this idea is carried out to an excessive degree or without discrimination, it may be noticed that the weight curve declines and the excretion of nitrogen increases. The same amount of fat that is well borne by one patient may be excessive for a second. The examination of the fæces enables one to avoid such errors, for the moment that fats appear in the fæces beyond the normal amount and in such forms as to indicate that they are not utilized, loss of weight and increased waste of nitrogen are imminent and may be anticipated by the coprologic balance.

Many examples might be cited of the value of coprologic examinations for the nutritive value of food depends upon the coefficient of intestinal utilization, somewhat characteristic of each individual but also varying according to the present condition of the digestive glands. The coprologic methods enable the physician to give foods easily digested by the particular individual, to push them up to the limit of assimilation, to reduce them in case of failure of utilization and thus prevent an excessive demand upon a failing function, and, in short to establish a rational dietary for the individual case, in accordance with the general principle that one lives, not by what he ingests but by what he digests.

40 rue de la Bienfaisance, Paris.

---

Lime Deposits In Skin. Darier and Brocq recognize the following forms: 1, Concrements in senile individuals; 2, true osteoma; 3, various tumors that have undergone secondary calcification; 4, subcutaneous granulomata ditto; 5, subcutaneous phleboliths. Ballot and Nahan, *Bull et Mem. de la Soc. de Radiologie Med. de Paris,* March, 1913, emphasize the value of X-rays in locating small lesions and state that gouty deposits are translucent to X-rays. (Note—We are inclined to believe that old gouty tophi, especially in the chalk regions of England, contain lime and hence would presumably cast a shadow.)

